# *Plasmodium falciparum* Hsp70-z, an Hsp110 homologue, exhibits independent chaperone activity and interacts with Hsp70-1 in a nucleotide-dependent fashion

**DOI:** 10.1007/s12192-016-0678-4

**Published:** 2016-02-19

**Authors:** Tawanda Zininga, Ikechukwu Achilonu, Heinrich Hoppe, Earl Prinsloo, Heini W. Dirr, Addmore Shonhai

**Affiliations:** 1 Department of Biochemistry, University of Venda, Private Bag X5050, Thohoyandou, 0950 South Africa; 2 Protein Structure-Function Research Unit, School of Molecular and Cell Biology, University of the Witwatersrand, Johannesburg, 2050 South Africa; 3 Department of Biochemistry and Microbiology, Rhodes University, P.O. Box 94, Grahamstown, 6140 South Africa; 4 Biotechnology Innovation Centre, Rhodes University, P.O. Box 94, Grahamstown, 6140 South Africa

**Keywords:** Malaria, *Plasmodium falciparum*, PfHsp70-z, Nucleotide exchange factor, Chaperone, Protein aggregation

## Abstract

The role of molecular chaperones, among them heat shock proteins (Hsps), in the development of malaria parasites has been well documented. Hsp70s are molecular chaperones that facilitate protein folding. Hsp70 proteins are composed of an N-terminal nucleotide binding domain (NBD), which confers them with ATPase activity and a C-terminal substrate binding domain (SBD). In the ADP-bound state, Hsp70 possesses high affinity for substrate and releases the folded substrate when it is bound to ATP. The two domains are connected by a conserved linker segment. Hsp110 proteins possess an extended lid segment, a feature that distinguishes them from canonical Hsp70s. *Plasmodium falciparum* Hsp70-z (PfHsp70-z) is a member of the Hsp110 family of Hsp70-like proteins. PfHsp70-z is essential for survival of malaria parasites and is thought to play an important role as a molecular chaperone and nucleotide exchange factor of its cytosolic canonical Hsp70 counterpart, PfHsp70-1. Unlike PfHsp70-1 whose functions are fairly well established, the structure-function features of PfHsp70-z remain to be fully elucidated. In the current study, we established that PfHsp70-z possesses independent chaperone activity. In fact, PfHsp70-z appears to be marginally more effective in suppressing protein aggregation than its cytosol-localized partner, PfHsp70-1. Furthermore, based on coimmunoaffinity chromatography and surface plasmon resonance analyses, PfHsp70-z associated with PfHsp70-1 in a nucleotide-dependent fashion. Our findings suggest that besides serving as a molecular chaperone, PfHsp70-z could facilitate the nucleotide exchange function of PfHsp70-1. These dual functions explain why it is essential for parasite survival.

## Introduction

Heat shock proteins (Hsps) play an important role in the development of the main agent of malaria, *Plasmodium falciparum*. Six members of the Hsp70 family of proteins are expressed by *P. falciparum* (Shonhai et al. 2007). Of these, two occur in the cytosol: PfHsp70-z/PfHsp110c and PfHsp70-1 (Shonhai et al. 2007; Muralidharan et al. 2012). PfHsp70-1 is a well-characterized canonical Hsp70 involved in prevention of protein aggregation and facilitates protein folding (Shonhai et al. 2008). PfHsp70-z was previously shown to be an essential protein implicated in the folding of proteins possessing asparagine-rich repeats (Muralidharan et al. 2012). Furthermore, we previously demonstrated that the protein is heat-induced and fairly stable against heat stress, suggesting that it plays an important role in the cytoprotection of malaria parasites against hostile conditions prevailing in the human host (Zininga et al. 2015a). Furthermore, the protein exhibits ATPase function and appears to occur as a dimer (Zininga et al. 2015a). However, apart from its proposed function as a chaperone based on studies in *P. falciparum* parasites, evidence for the direct function of this protein in protein quality control remains to be demonstrated.

Hsp70 proteins are composed of an N-terminal nucleotide binding domain (NBD), which confers them with ATPase activity and a C-terminal substrate binding domain (SBD). In the ADP-bound state, Hsp70 possesses high affinity for substrate and releases the folded substrate when it is bound to ATP. The two domains are connected by a linker segment. Hsp110 proteins possess an extended lid segment, a feature that distinguishes them from canonical Hsp70s. *P. falciparum* Hsp70-z (PfHsp70-z) is a member of Hsp110 family of Hsp70-like proteins. Hsp110s are known to inhibit protein aggregation through their role as holdases of misfolding proteins (Goeckeler et al. 2002). For a long time, the role of Hsp110 was poorly understood until a study by Dragovic et al. (2006) reported that yeast Hsp110 (Sse1p) and human Hsp110 (HSPH1) could serve as nucleotide exchange factors (NEFs) of their respective canonical Hsp70 counterparts.

Although Hsps are generally conserved across species, it is known that some of them exhibit distinct functional features across species (Shonhai et al. 2007; Gitau et al. 2012). In addition, the distribution of co-chaperones (molecules that regulate) and the chaperone role of Hsps tend to vary between species, thereby making the functions of these apparently conserved molecules unique across species and within subcellular compartments (Botha et al. 2007; Zininga and Shonhai 2014). Hsps are also implicated in the development of malaria parasites and are implicated in protein trafficking and virulence of the disease (Shonhai et al. 2011; Külzer et al. 2012). In light of the above aspects, it is important to study the role of Hsps in the context of the conserved but also fairly divergent role across species. For example, in spite of their conservation, Hsp70 and Hsp90 have been proposed as potential antimalarial drug targets (Shonhai 2010; Cockburn et al. 2011; Shahinas et al. 2013).

PfHsp70-z has been predicted to serve as an NEF of PfHsp70-1 (Shonhai et al. 2007), although this remains to be experimentally validated. In human cells, nucleotide exchange function of cytosol-localized Hsp70s is mediated by several NEFs such as Bcl2-associated athanagene-1 (Bag-1) and heat shock protein binding protein 1 (HspBP1) (Sondermann et al. 2001; Shomura et al. 2005) in *P. falciparum* PfHsp70-z appears to be the sole NEF of PfHsp70-1 (Zininga et al. 2015a). Nucleotide exchange indirectly determines the substrate dwell time on the Hsp70_SBD_ thereby influencing substrate fate (Mandal et al. 2010) as the premature release of substrates from Hsp70 could result in their aggregation, leading to their degradation (Mayer and Bukau 2005). PfHsp70-z is thought to be the sole NEF of PfHsp70-1 (Zininga et al. 2015a). In light of this, its importance in the survival of malaria parasites could not be overemphasized.

In the current study, we investigated the interaction between PfHsp70-z and its cytosolic counterpart, PfHsp70-1. We further investigated the independent chaperone function of PfHsp70-z. Our findings suggest that PfHsp70-z interacts with PfHsp70-1 in a nucleotide-dependent fashion. Furthermore, we established that although ATP promoted its interaction with PfHsp70-1, the chaperone function of PfHsp70-z was not regulated by nucleotides. We surmise that PfHsp70-z is involved in a broad spectrum of functions to maintain proteostasis through its chaperone role and possibly serves as a NEF of PfHsp70-1. Bioinformatics-based evidence revealed that Hsp110 proteins are highly conserved in plasmodial species. However, the proteins exhibit some distinct structural features from yeast and human homologues based on sequence alignment. In light of its essential role in *P. falciparum* and its unique functional features, PfHsp70-z may represent a potential antimalarial drug target. In addition, the biochemical assays for the chaperone function of PfHsp70-z we established in this study may pave way for the high throughput selection of antimalarial inhibitors of PfHsp70-z.

## Materials and methods

### Materials

Nickel nitrilotriacetic acid (NTA) resin and enhanced chemiluminescence (ECL) were purchased from Thermo Scientific (USA). Mouse horseradish peroxidase (HRP)-conjugated monoclonal anti-polyhistidine (His) antibodies were purchased from Sigma-Aldrich, (USA). The anti-PfHsp70-z and anti-PfHsp70-1 antibodies which we previously described (Shonhai et al. 2008; Zininga et al. 2015a) were used in the current study. Chemical reagents used in this study were purchased from Merck Chemicals (Darmstadt, Germany), Thermo Scientific (IL, USA), Melford (Suffolk, UK), and Sigma-Aldrich (USA), unless otherwise mentioned.

### Bioinformatics analysis of PfHsp70-z

The protein sequence of PfHsp70-z was aligned to sequences of other cytosolic Hsp110 homologues from *Plasmodium* species retrieved from the PlasmoDB data base (Plasmodb.org). The PfHsp70-z sequence was also aligned to sequences of Hsp110s from *Homo sapiens*, *Saccharomyces cerevisiae*, and *Mus musculus* that were retrieved from the NCBI (http://www.ncbi.nlm.nih.gov). The following Hsp110 protein sequences were retrieved (accession numbers are provided): *P. falciparum*, PfHsp110/PfHsp70-z (PF3D7_0708800); *Plasmodium vivax*, PvHsp110 (PVX_087970); *Plasmodium knowlesi*, PkHsp110 (PKH_010690); *Plasmodium chabaudi*, PcHsp110 (XP_745506.1); *Plasmodium berghei*, PbHsp110 (PBANKA_121930); *Plasmodium yoelii*, PyHsp110 (PYYM_1222000); *Plasmodium cynomolgi*, PcyHsp110 (PCYB_011590); *H. sapiens*, HSPH1 (NP_006635.2); *M. musculus*, mHsp110 (NP_038587); *S. cerevisiae*, Sse1 (Q875V0); and *S. cerevisiae*, Sse2 (CAA85130.1). The retrieved protein sequences were analyzed using MAFFT version 7 (http://www.ebi.ac.uk/Tools/msa/mafft/) and Boxshade (http://www.ch.embnet.org/software/BOX_form.html).

### Overexpression and purification of PfHsp70-z

A codon-harmonized form of the full length *PfHsp70-z* (PlasmoDB accession no. PF3D7_088000) which we previously described was used for the expression of recombinant PfHsp70-z protein using *Escherichia coli* JM109 cells following a previously described method (Zininga et al. 2015a). The purified protein was extensively dialyzed overnight at 4 °C against a storage buffer (10 mM Tris, pH 7.5, 150 mM NaCl, 0.8 mM DTT, 10 % (*v*/*v*) glycerol). Protein concentrations were estimated by Bradford assay. The presence of PfHsp70-z was confirmed using both rabbit anti-His, rabbit polyclonal peptide anti-PfHsp70-z antibodies, and HRP-conjugated anti-rabbit IgG secondary antibodies (1:2000) (ThermoScientific, USA). Imaging of the protein bands on the blot was conducted using the ECL kit as per manufacturer’s instructions. Images were captured using ChemiDoc Imaging system (Bio-Rad, USA).

### Overexpression and purification of PfHsp70-1 and PfHsp70-1 nucleotide binding domain

A construct expressing PfHsp70-1 (pQE30/PfHsp70-1) was used for the expression of recombinant PfHsp70-1 protein using *E. coli* XL1 Blue cells following a previously described method (Shonhai et al. 2008). Another construct expressing the nucleotide binding domain of PfHsp70-1 (PfHsp70-1_NBD_) (Zininga et al. 2015b) was used to express this subdomain of the protein. The recombinant proteins were purified as previously described (Zininga et al. 2015b).

### Determination of the nucleotide binding affinity of PfHsp70-z

The assay was conducted at room temperature (25 °C) using a Bio-Rad ProteOn XPR36 system. Filter sterilized and degassed PBS-Tween (4.3 mM Na_2_HPO_4_, 1.4 mM KH_2_PO_4_, 137 mM NaCl, 3 mM KCl, 0.005 % (*v*/*v*) Tween 20, and 20 mM EDTA; pH 7.4) was used as running buffer. PfHsp70-1 and PfHsp70-z (as ligands) were immobilized at concentrations of 0.5 and 1 μg/ml, and PfHsp70-1_NBD_ (as ligand) was immobilized as 1 μg/ml. At these concentrations, we achieved 197 response units (RU) for PfHsp70-z, 198 for PfHsp70-1, and 180 for PfHsp70-1_NBD_ RU per immobilization surface. The immobilization of ligands on the HTE chip surface was achieved through the Tris-NTA complex activated with Ni^2+^, to enable stable binding of polyhistidine-tagged proteins following a protocol that was provided by the manufacturer (Bio-Rad, USA). As analytes, aliquots of ATP/ADP were prepared at final concentration of 1.25, 2.50, 5, 10, and 20 nM and were injected at 100 μl/min in each horizontal channel. Association was allowed for 2 min and dissociation was monitored for 8 min. Steady-state equilibrium constant data was processed and analyzed using ProteOn Manager software version 3.1.0.6.

### Investigation of the effects of nucleotides on the conformation of PfHsp70-z by partial proteolysis

Nucleotide-dependent conformational alterations of PfHsp70-z were investigated by partial trypsin proteolysis. Briefly, 0.45 μg/ml recombinant PfHsp70-z was incubated in the absence and presence of 5 mM nucleotide ATP/ADP in PBS (137 mM NaCl, 2.7 mM KCl, 4.3 mM Na_2_HPO_4_, and 1.4 mM KH_2_PO_4_) for 10 min at 30 °C. The proteolytic reaction was started by addition of 0.25 ng/ml of trypsin. Samples were taken at 0, 5, 15, and 30 min time points and added to 4 × sodium dodecyl sulfate (SDS) loading buffer (0.5 M Tris–HCl, pH 6.8, 10 % (*v*/*v*) glycerol, 10 % (*w*/*v*) SDS, 5 % (*v*/*v*) β-mercaptoethanol, 1 % (*w*/*v*) bromophenol blue) and analyzed using sodium dodecyl sulfate polyacrylamide gel electrophoresis (SDS-PAGE) and stained using the Pierce silver stain kit. Western analysis was conducted to verify the authenticity of recombinant (His)_6_-PfHsp70-z using α-His antibodies (1:200). Imaging of the protein bands on the blot was conducted using the ECL kit as per manufacturer’s instructions. The images were captured using ChemiDoc Imaging System (Bio-Rad, USA).

### Investigation of the nucleotide-dependent conformational changes of PfHsp70-z using tryptophan fluorescence-based analysis

Nucleotide-dependent conformational alterations of PfHsp70-z were investigated by tryptophan fluorescence. Recombinant PfHsp70-z (0.45 μg/ml) was incubated in the absence and presence of 5 mM nucleotide ATP/ADP in assay buffer A (25 mM 4-(2-hydroxyethyl)-1-piperazineethanesulfonic acid (HEPES)-potassium hydroxide (KOH) pH 7.5, 100 mM KCl, 10 mM MgOA_c_) for 10 min at 20 °C. Time-course was conducted for 65 min by measuring fluorescence from different PfHsp70-z recombinant proteins incubated in buffer A. Photobleaching was minimized by subjecting the samples to fluorescence excitation once. Fluorescence was measured between 320 and 450 nm after initial excitation at 295 nm using a JASCO FP-6300 spectrofluorometer. Relative fluorescence was calculated as the average value obtained from at least seven spectrum scans less the baseline (buffer with or without nucleotides in the absence of protein) reading.

### Protein aggregation suppression assay

The chaperone function of recombinant PfHsp70-z was investigated by analyzing its ability to prevent heat-induced aggregation of luciferase from *Photinus pyralis* (Sigma-Aldrich) using spectrophotometry, following a previously described protocol (Polier et al. 2010) with minor modifications. The assay was initiated by adding 0.15 μM luciferase and 0.2 μM recombinant chaperone in assay buffer B (25 mM HEPES-KOH, pH 7.5, 5 mM MgCl, 5 mM NaOA_C_, 50 mM KCl, 5 mM β-mercaptoethanol) heated to 48 °C. Protein aggregation was monitored for 60 min as change in absorbance readings at 360 nm using the SpectraMax M3 spectrometer (Molecular Devices, USA). The aggregation value was determined as a proportion of luciferase aggregation set as 100 %. To determine if the chaperone function was dependent on the presence of nucleotides, 5 mM ATP/ADP was added to the reaction mixture 15 min from the onset of the reaction.

In addition, the chaperone function of PfHsp70-z was investigated by monitoring the heat-induced aggregation of another model protein, malate dehydrogenase (MDH) from *porcine* heart (Sigma-Aldrich) following previously described protocols (Shonhai et al. 2008; Luthuli et al. 2013; Makumire et al. 2014) with minor modifications. The assay was initiated by adding 0.25 μM MDH and 0.2 μM recombinant PfHsp70-z in assay buffer C (50 mM Tris–HCl, pH 7.5, 100 mM NaCl), and reaction mix was incubated at 48 °C. Protein aggregation values were determined as previously described.

### Investigation of the direct association of PfHsp70-z with PfHsp70-1 coaffinity chromatography

*P. falciparum* 3D7 cells were cultured as previously described (Zininga et al. 2015a, b). Sorbitol synchronized parasites were harvested at the trophozoite stage from two fractions, one consisting of parasites that had been subjected to heat shock at 42 °C for 2 h prior to harvesting and the other from parasites that were maintained under normal temperature conditions (37 °C). Parasites were collected by centrifugation at 5000×*g* for 10 min after (0.1 %) saponin lysis of erythrocytes followed by an extensive wash step using PBS (pH 7.4). The parasite lysates were resuspended in 500 μl of lysis buffer (25 mM Tris–HCl pH 7.5, 150 mM NaCl, 1 mM EDTA, 1 % (*v/v*) Tween-20, and containing 1 mM PMSF). Parasite lysate containing approximately 300 μg of total protein was suspended in a protein A/G magnetic resin to which anti-PfHsp70-z antibodies had been attached (Pierce, Thermo Scientific). As a control, beads without α-PfHsp70-z attached were used. Binding was allowed to occur for 2 h at room temperature (25 °C) with gentle agitation. To investigate the effect of nucleotide on PfHsp70-z binding to PfHsp70-1, the suspension was split into three aliquots. One aliquot was adjusted to 5 mM ATP, the other to 5 mM ADP, and the other no nucleotides were added. Following subsequent washing steps using Tris-buffered saline (TBS) wash buffer (25 mM Tris–HCl pH 7.5, 500 mM NaCl, and 0.05 % Tween 20), 150 μl of elution buffer (100 mM glycine, pH 2.5) was added. This was followed neutralization using 100 mM Tris pH 7.5. The precipitate was analyzed by Western blot technique using anti-PfHsp70-1 antibodies.

In addition to the immunoaffinity chromatography assay, the direct association of PfHsp70-z and PfHsp70-1 was investigated using recombinant PfHsp70-z immobilized onto polyhistidine resin to attract PfHsp70-1 from the parasite lysate. Parasites released upon saponin lysis of the erythrocytes were resuspended in Pierce lysis buffer (Thermo Scientific). The parasite lysate (prey) was mixed with the purified polyhistidine-tagged recombinant PfHsp70-z (bait) which was immobilized on HisPur Cobalt resin (Thermo Scientific). Binding was allowed for 4 h at 4 °C. As a control, beads without immobilized recombinant PfHsp70-z protein were used. The samples were washed extensively using TBS/Pierce lysis buffer. The bait-prey proteins were subsequently eluted in 250 μl elution buffer (TBS/Pierce lysis buffer, 290 mM imidazole). To investigate the effect of nucleotide on the interaction between PfHsp70-z and PfHsp70-1, the suspension was split into three aliquots. One aliquot was adjusted to 5 mM ATP, the second to 5 mM ADP, and no nucleotides were added to the third. The eluates were analyzed by Western blot analysis using anti-PfHsp70-1 antibodies.

### Assessment of the interaction between PfHsp70-z and PfHsp70-1 using slot blot analysis

Purified recombinant PfHsp70-z (2, 8, 16 μg) and the negative control, 16 μg of bovine serum albumin (BSA) were spotted onto nitrocellulose membrane using Bio-Dot SF apparatus connected to a vacuum pump (Sultana and Butterfield 2008). The membrane was blocked with 5 % (*w*/*v*) fat-free milk in TBST (50 mM Tris–HCl pH 7.5, 150 mM NaCl, 0.1 % (*v*/*v*) Tween 20) and overlaid with either PfHsp70-1/PfHsp70-1_NBD_ (10 μg/ml) in the presence of 5 mM ATP/ADP and incubated overnight at 4 °C, followed by three washes with TBST for 15 min. The nucleotide concentrations were maintained in the subsequent steps of the protocol. Protein was detected by Western blot technique using rabbit raised polyclonal α-PfHsp70-1 antibody (1:2000) (Shonhai et al. 2008). Monoclonal HRP-conjugated α-rabbit IgG (1:4000) (Sigma-Aldrich, USA) was used as secondary antibody. Imaging of the protein bands on the blot was conducted using the ECL kit as per manufacturer’s instructions. The images were captured using ChemiDoc imaging system (Bio-Rad, USA).

### Analysis of the interaction between PfHsp70-z and PfHsp70-1 using surface plasmon resonance

The assay was conducted using Bio-Rad ProteOn XPR36 surface plasmon resonance (SPR) system. Filter sterilized and degassed PBS-Tween (4.3 mM Na_2_HPO_4_, 1.4 mM KH_2_PO_4_, 137 mM NaCl, 3 mM KCl, 0.005 % (*v*/*v*) Tween 20, and 20 mM EDTA; pH 7.4) was used as running buffer. The immobilization of ligands was achieved through covalent attachment to the modified alginate polymer layer on the GLC sensor chip (Bio-Rad, USA) via amine coupling following a protocol that was provided by the manufacturer. PfHsp70-1 and PfHsp70-z (as ligands) were immobilized at concentrations of 0.5 and 1 μg/ml, and PfHsp70-1_NBD_ (as ligand) was immobilized as 1 μg/ml. At these concentrations, we achieved 187 RU for PfHsp70-z, 196 for PfHsp70-1, and 198 for PfHsp70-1_NBD_ RU per immobilization surface. As analytes, aliquots of PfHsp70-z, PfHsp70-1, and PfHsp70-1_NBD_ were prepared at final concentration of 125, 250, 500, 1000, and 2000 nM and were injected at 50 μl/min in each horizontal channel. Association was allowed for 2 min, and dissociation was monitored for 10 min. The assay was conducted at room temperature (25 °C). The final data was determined by subtracting the baseline RU (buffer containing ATP/ADP without analyte protein). The association rate constant, dissociation rate constant, and equilibrium constant data were processed and analyzed using ProteOn Manager software version 3.1.0.6 involving concatenating the responses of the analyte at variable concentrations.

## Results

### Plasmodial Hsp110 proteins are conserved

**Fig. 1:**
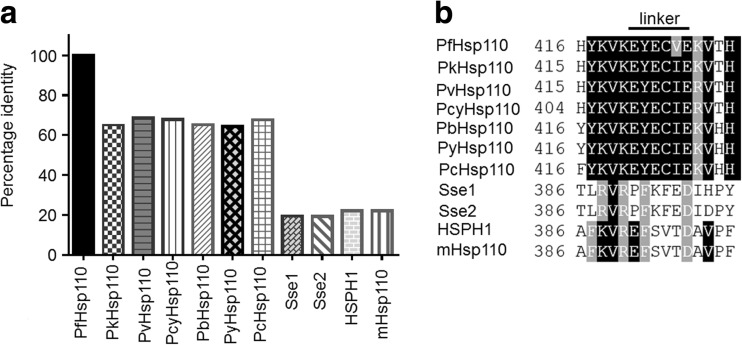
Hsp110 protein sequence identities and alignment of linker residues. Multiple sequence alignment of Hsp110s from the following organisms was conducted: *P. falciparum*, PfHsp110; *P. vivax*, PvHsp110; *P. knowlesi*, PkHsp110; *P. chabaudi*, PcHsp110; *P. berghei*, PbHsp110; *P. yoelii*, PyHsp110; *P. cynomolgi*, PcyHsp110; *Homo sapiens*, HSPH1; *Mus musculus*, mHsp110; *Saccharomyces cerevisiae*, Sse1; and *S. cerevisiae*, Sse2. The percentage identities of the sequences to PfHsp70-z sequence are depicted in bar graphs (**a**). A sequence alignment of residues constituting the linker is shown (**b**)

A fairly high degree of homology in the cytosolic Hsp110 proteins was observed across the plasmodial species in comparison to their human and yeast counterparts (Fig. 1a). Based on sequence information, PfHsp70-z shares the highest sequence identity with its *P. vivax* homologue (68.5 %). On the other hand, PfHsp70-z exhibited a lower degree of conservation when compared to its human and mouse homologue Hsp110 (sequence identities of 22.2 and 22.2 %, respectively) and its yeast homologues Sse1 and Sse2 (19.5 and 19.4 %, respectively). Hsp110 residues that are known to be important for the interaction with Hsp70 (data not shown) (Schuermann et al. 2008) are more highly conserved in the Hsp110_NBD_ compared to those in the Hsp110_SBD_ (data not shown). It is known that in the ATP-bound form, Hsp110 binds Hsp70 stably (Andreasson et al. 2008). Interestingly, residues in Hsp110-Hsp70 association appear poorly conserved across the Hsp110 homologues (data not shown). On the other hand, the Hsp110 residues implicated in ATP binding (Moran et al. 2013) are well conserved across the Hsp110 homologues.

PfHsp70-z possesses an N-terminal NBD (PfHsp70-z_NBD_) composed of residues 1–420, a putative linker region at position 421–426, and a SBD composed of SBDβ residues 427–660, and SBDα residues 661–729 (Fig. 1b). The proposed linker of the cytosolic Hsp110s from plasmodial species depicted by the sequence, EYECVE; is fairly distinct from that of yeast (PFKFED) and human/mouse Hsp110 (EFSVTD) (Fig. 1b). By comparison, the linker region of the canonical Hsp70s, however, is well conserved across species (Shonhai et al. 2007). This suggests that the linker of Hsp110 could play an important regulatory role that uniquely defines the functional characteristics of this family of proteins.

### Overexpression and purification of recombinant PfHsp70-z, PfHsp70-1, and PfHsp70-1_NBD_

**Fig. 2:**
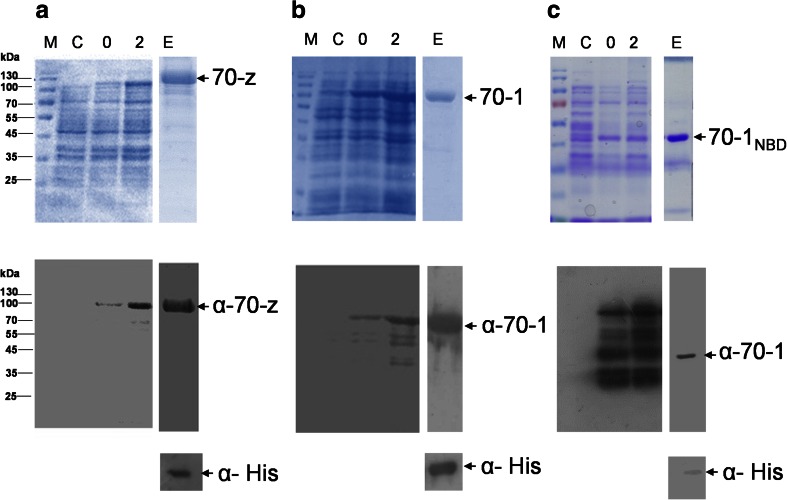
Expression and purification of recombinant forms of PfHsp70-z, PfHsp70-1, and PfHsp70-1_NBD_. PfHsp70-z expressed in *E. coli* JM109 cells transformed with construct expressing PfHsp70-z and PfHsp70-1/PfHsp70-1_NBD_ was expressed in *E. coli* XL1 Blue cells. SDS-PAGE (12 %) and Western blot images representing the expression and purification of recombinant forms of PfHsp70-z (**a**) (70-z), PfHsp70-1 (**b**) (70-1), and PfHsp70-1_NBD_ (**c**) (70-1_NBD_). *Lane M*: Page ruler (Thermo Scientific) in kilodalton is shown on the *left hand side. Lane C*: The total extract for cells transformed with a neat pQE30 plasmid. *Lane 0*: Total cell extract transformed with pQE30/PfHsp70-z, pQE30/PfHsp70-1, and pQE30/PfHsp70-1_NBD_ prior to IPTG induction. *Lane 2*: Total cell lysate obtained 2 h post-induction. *Lane E*: Protein eluted from Ni^2+^ chelate affinity matrix using 500 mM imidazole. *Lower panels*: Western blot confirming expression and purification of PfHsp70-z, PfHsp70-1, and PfHsp70-1_NBD_

We expressed and purified the recombinant forms of PfHsp70-z, PfHsp70-1, and PfHsp70-1_NBD_ using previously described protocols (Shonhai et al. 2008; Zininga et al. 2015a, b). We validated the authenticity of recombinant PfHsp70-z by Western blot using an antibody raised against a unique peptide segment of the protein and anti-polyhistidine antibodies (Fig. 2a). Similarly, full length PfHsp70-1 was overexpressed and purified from *E. coli* XL1-Blue cells (Fig. 2b). Furthermore, the truncated PfHsp70-1 (PfHsp70-1_NBD_) was overexpressed and purified from *E. coli* XL1 Blue cells (Fig. 2c). Western blotting analysis using antibodies raised against PfHsp70-1 as well as antibodies that recognize the polyhistidine-tag, we detected both the full length PfHsp70-1 and the truncated version PfHsp70-1_NBD_ (Fig. 2b, c, lower panels). The specificity of the antibody raised against the select peptide sequence of PfHsp70-z was previously reported (Zininga et al. 2015a). The peptide-specific antibody binds PfHsp70-z without recognizing other Hsp70s including its plasmodial homologue, PfHsp70-1 (Zininga et al. 2015a).

### Nucleotide equilibrium binding assay

**Fig. 3:**
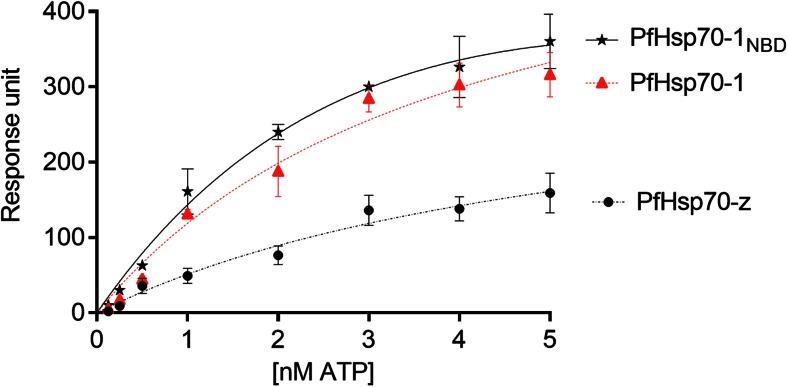
Equilibrium analysis of ATP binding by PfHsp70-z and PfHsp70-1/PfHsp70-1_NBD_. The data represents the equilibrium analysis of ATP binding constants for all three proteins. The equilibrium constant *K*_D_ (Table 1) was obtained for PfHsp70-z, PfHsp70-1, and PfHsp70-1_NBD_

Affinities for the binding of nucleotides to PfHsp70-z, relative to PfHsp70-1 and its truncated version (PfHsp70-1_NBD_), were determined using SPR analysis. The kinetics representing binding of the nucleotides to the respective proteins at equilibrium binding were determined (Fig. 3). The *K*_D_ values for ATP binding to PfHsp70-1 and its truncated form, PfHsp70-1_NBD_, were comparable in magnitude (approximately 3.5 μM) (Table 1). PfHsp70-z exhibited *K*_D_ values for ATP binding which were higher by approximately one order of magnitude (25.3 μM) compared to data obtained for PfHsp70-1/PfHsp70-1_NBD_. These data suggest that although PfHsp70-1 and PfHsp70-z both exhibit high affinity (in the micro molar range) for ATP, however, PfHsp70-1 binds ATP with much higher affinity. Since ATP binds to full length PfHsp70-1 and its truncated form (PfHsp70-1_NBD_) with comparable affinity, this demonstrates that in the absence of bound substrate, the SBD of PfHsp70-1 has little influence on the steady-state ATP binding kinetics.

**Table 1:** Comparative affinities for ATP binding to PfHsp70-z and PfHsp70-1

Protein	ATP
	*K*_D_ (μM) [±standard deviation]
PfHsp70-z	25.3 (±1.1)
PfHsp70-1	3.48 (±0.4)
PfHsp70-1_NBD_	3.47 (±0.2)

The standard deviations shown in parenthesis were obtained from at least three independent assays

### ATP induces conformational changes to PfHsp70-z

**Fig. 4:**
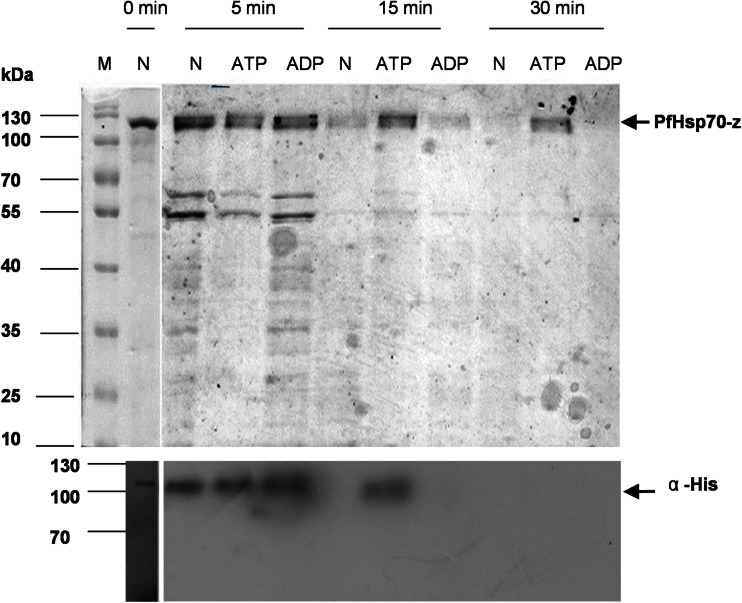
Limited proteolysis confirming nucleotide-induced conformational changes in PfHsp70-z. *Upper panel*: Silver-stained SDS-PAGE gel (12 %) representing the partial tryptic digestion of recombinant PfHsp70-z in nucleotide-free buffer (*N*) and in the presence of 5 μM ADP/ATP. *Lower panel*: Western blot representing PfHsp70-z fragments that were detected using α-His_6_ antibody. PfHsp70-z was digested at 30 °C using 0.25 ng/ml trypsin, and samples were collected at the indicated time points

We subjected recombinant PfHsp70-z protein to partial proteolysis in the absence of nucleotide and in presence of ATP/ADP to investigate the effect of the nucleotides on the conformation on the protein. Profiles of fragments that were generated upon the proteolysis of PfHsp70-z in the presence of ADP and in the absence of nucleotide were similar. After 30 min, both nucleotide-free and ADP-bound forms of PfHsp70-z were completely digested. Overall, this suggests that the ADP-bound form of PfHsp70-z may assume a conformation that closely resembles that of the nucleotide-free form of the protein (Fig. 4). However, the proteolysis of the ATP-bound PfHsp70-z generated digestion fragments that were unique and part of the protein remained undigested after 30 min. However, the fragment that remained after 30 min could not be detected by Western blot analysis suggesting that it may have lost its N-terminal polyhistidine-tag. Altogether, the findings suggest that ATP induced structural changes to PfHsp70-z that resulted in a more compact conformation with increased resilience to tryptic digestion.

**Fig. 5:**
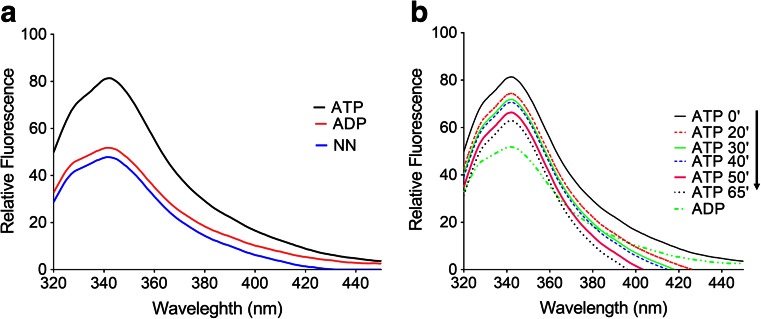
The conformation of PfHsp70-z is regulated by nucleotides. Tryptophan fluorescence signals obtained upon incubating PfHsp70-z in the absence of nucleotide or in the presence of 5 μM ATP/ADP (**a**). A time-course assessment of ATP on fluorescence intensity of PfHsp70-z was conducted (**b**). The time-course was conducted on samples collected at the indicated time points

PfHsp70-z possesses two tryptophan residues at positions Trp436 and Trp690. Taking advantage of this, we previously demonstrated that recombinant PfHsp70-z exhibited a shift in fluorescence when it was treated by various levels of urea (Zininga et al. 2015a). In the current study, we employed the same approach to gain further insight into the conformational states of PfHsp70-z in nucleotide-free buffer and in the presence of ADP/ATP. Data from the tryptophan-based fluorescence analysis suggested that nucleotide-free and ADP-bound forms of the protein may exhibit closely related conformational states (Fig. 5a). On the other hand, the ATP-bound form of the protein assumed a unique conformation as confirmed by tryptophan fluorescence analysis.

We further incubated PfHsp70-z for at least 65 min in the presence of ATP, followed by collection of samples at different time points and subsequent fluorescence analysis. A parallel set of control samples was analyzed to account for the spontaneous photo-bleaching of tryptophan upon exposure to the excitation wavelength. We observed conformational changes over the course of time. We surmised that the conformational changes may possibly reflect hydrolysis of ATP by PfHsp70-z as the fluorescence peak shifted towards that of its ADP-bound state over time (Fig. 5b). Altogether, these findings support the observations that we made using the limited proteolysis assay.

### PfHsp70-z suppresses heat-induced aggregation of luciferase and malate dehydrogenase

**Fig. 6:**
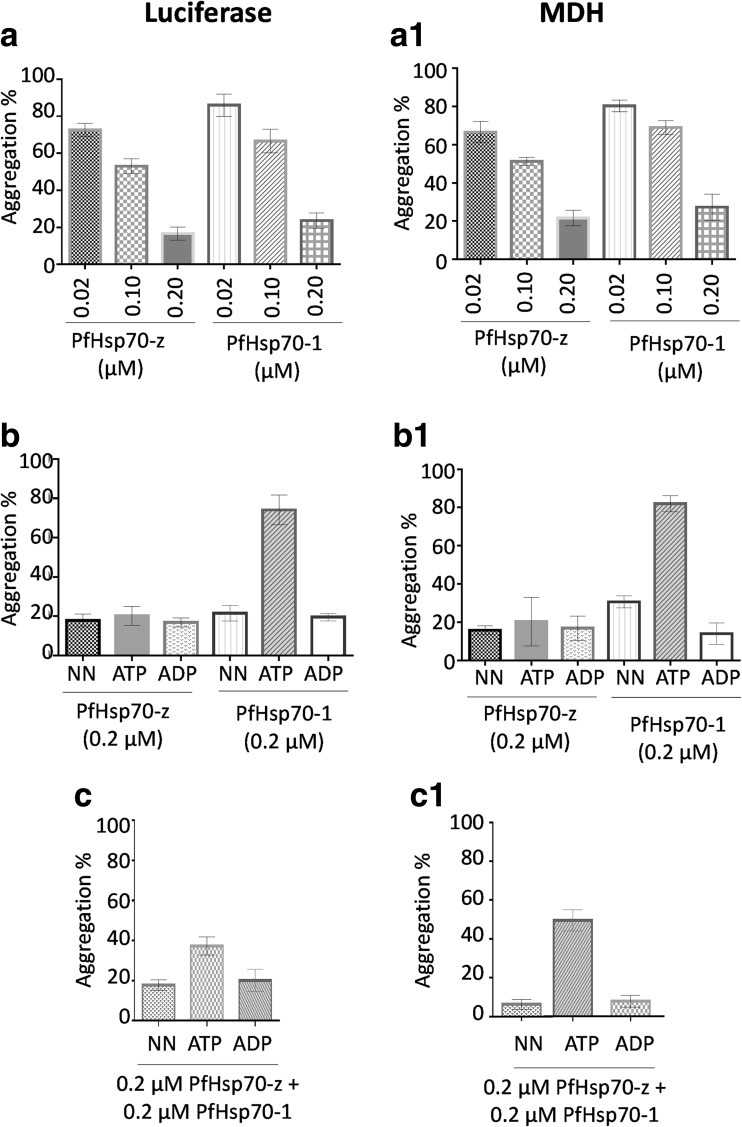
PfHsp70-z suppresses heat-induced aggregation of luciferase and MDH. The heat-induced aggregation of luciferase and MDH was assessed in vitro at 48 °C. Dose–response assessment of the independent capabilities of PfHsp70-z and PfHsp70-1 to suppress heat-induced aggregation of luciferase and MDH (*A*, *A1*); the assay was repeated in the presence of 5 μM ADP/ATP (*B*, *B1*). The activity of PfHsp70-1 and PfHsp70-z in the presence of ATP compared to the nucleotide-free state and ADP-bound state was statistically significant (*p* < 0.005), respectively. The assay was repeated to assess the activity for the combination of PfHsp70-z and PfHsp70-1 (*C*, *C1*), under various conditions: nucleotide-free buffer (*NN*) and presence of ADP/ATP, respectively. Standard deviations obtained from three replicate assays are shown

The independent chaperone activity of recombinant PfHsp70-z was determined by assessing its ability to prevent heat-induced aggregation of luciferase. Based on this assay, protein aggregation results in increased turbidity which is monitored by taking absorbance readings at 360 nm. First, it was important to establish that PfHsp70-z was heat-stable. The protein did not aggregate at 48 °C (data not shown). This is in line with our previous observation that PfHsp70-z is stable for up to 80 °C (Zininga et al. 2015a). We then compared the capabilities of PfHsp70-z and PfHsp70-1 to suppress heat-induced aggregation of proteins in vitro. PfHsp70-z suppressed the aggregation of both luciferase (Fig. 6A) and MDH (Fig. 6A1) in a concentration-dependent fashion. The optimum chaperone activities for PfHsp70-z and PfHsp70-1 were observed at a 1:1 chaperone-to-substrate ratio (Fig. 6A, A1). The protein aggregation suppression function of the canonical cytosolic Hsp70 (PfHsp70-1) has been previously confirmed (Shonhai et al. 2008). Based on our findings, PfHsp70-z is marginally more effective in suppressing protein aggregation compared to PfHsp70-1.

We repeated the protein aggregation assays in the presence of ATP or ADP and found that the chaperone function of PfHsp70-z was not influenced by nucleotide (Fig. 6B, B1). On the other hand, as previously observed (Shonhai et al. 2008), the addition of ATP to the assay mix inhibited the protein aggregation suppressive function of PfHsp70-1 (Fig. 6C, C1). However, ADP did not affect the chaperone function of PfHsp70-1. It is known that ADP enhances substrate binding (holdase) function of Hsp70 (Mayer et al. 2000), and therefore this finding is in agreement with this position for both PfHsp70-z and PfHsp70-1. However, in the presence of ATP, the activity of the combination of the two proteins was slightly reduced (Fig. 6C, C1). We attribute the poorer efficiency of the combined action of PfHsp70-z and PfHsp70-1 in the presence of ATP to the inhibition of PfHsp70-1 by ATP (Shonhai et al. 2008; Fig. 6C, C1).

### PfHsp70-z interacts with PfHsp70-1 in a nucleotide-dependent fashion

**Fig. 7:**
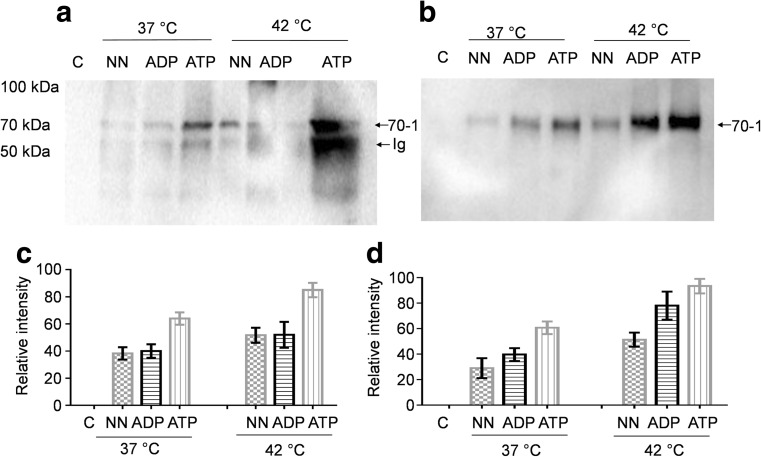
PfHsp70-z associates with PfHsp70-1. Pulldown assays conducted using antibodies against PfHsp70-z and recombinant PfHsp70-z protein. Prey proteins in parasite cell lysate harvested from cultures growing under normal temperature (37 °C) and heat stress (42 °C) conditions. Prey protein was allowed to bind onto columns on which the antibody (**a**) or recombinant PfHsp70-z (**b**) were immobilized, respectively. Binding was allowed to occur in the absence of nucleotide (*NN*) or presence of 5 μM ADP/ATP, respectively. The eluate was analyzed under reducing conditions and subsequently probed by Western blot using anti-PfHsp70-1 antibodies. The representation of the densitometric analyses for data is provided (*lower panels*; **c** and **d**, respectively). *Lane C*: control sample (conducted on column with beads minus anti-PfHsp70-z or PfHsp70-z recombinant protein). Cell lysate used in control assay was obtained from culture incubated at 37 °C. *Lanes 37 and 42 °C*: Immunoaffinity conducted using cell lysate obtained from cells cultured at the respective temperatures which was allowed to bind to beads onto which anti-PfHsp70-z (**a**) or recombinant PfHsp70-z (**b**) were immobilized, respectively. *Arrows* in panel **a** show bands representing PfHsp70-1 (70-1) and Ig heavy chain (*Ig*), respectively

PfHsp70-z and PfHsp70-1 are both reported to localize to the cytosol of the parasite (Pesce et al. 2008; Gitau et al. 2012; Muralidharan et al. 2012). Furthermore, both proteins are stress-induced and it is further thought that PfHsp70-z may serve as the sole NEF of PfHsp70-1 (Zininga et al. 2015a). For this reason, we investigated the physical association of these two molecular chaperones. Cell lysate harvested from blood stage parasites that were cultured under normal temperature (37 °C) and heat stress conditions (42 °C) was used to conduct immunoaffinity chromatography assays using anti-PfHsp70-z antibodies. As a control, we allowed the cell lysate to flow through a column that was packed with beads in the absence of immobilized anti-PfHsp70-z antibodies. There was no evidence for the presence of PfHsp70-1 protein in the control eluate (Fig. 7a, lane C). Next, the buffer in which the cell lysate was suspended was modified as follows: one batch had no supplemented nucleotide, and to the other two batches we added 5 mM ADP or ATP, respectively. Following this, we conducted immunoaffinity chromatography using a column onto which anti-PfHsp70-z antibodies were immobilized. We then probed for the presence of PfHsp70-1 associated with the PfHsp70-z protein. We previously validated that anti-PfHsp70-1 antibodies do not cross-react with PfHsp70-z (Zininga et al. 2015a), and this was an important requirement for this assay as antibody cross-reactivity would have confounded our investigation. We observed that higher titers of PfHsp70-1 protein were associated with PfHsp70-z in the parasites that were cultured at 42 °C compared to culture that was maintained at 37 °C (Fig. 7a). However, the association of PfHsp70-1 with PfHsp70-z was promoted in the presence of nucleotide, with ATP being more effective at promoting the association compared to ADP. This trend was observed for cell lysate obtained from cultures maintained both under normal and heat stress conditions. Altogether, these findings suggest that not only are both PfHsp70-z and PfHsp70-1 induced by heat stress but that their association is sustained under stressful conditions. Furthermore, their association is nucleotide-dependent.

As a follow-up to the coimmunoaffinity analysis, we conducted a coaffinity chromatography assay using polyhistidine-tagged recombinant PfHsp70-z protein to attract prey protein from the parasite lysate (Fig. 7b). As a control, we allowed the cell lysate to pass through the cobalt resin without recombinant PfHsp70-z protein immobilized. There was no evidence of the presence of PfHsp70-1 in the control eluate (Fig. 7b, lane C). Our findings using this assay mirror the observations we made based on the coimmunoaffinity assay further confirming that ATP enhances association of PfHsp70-z with PfHsp70-1.

**Fig. 8:**
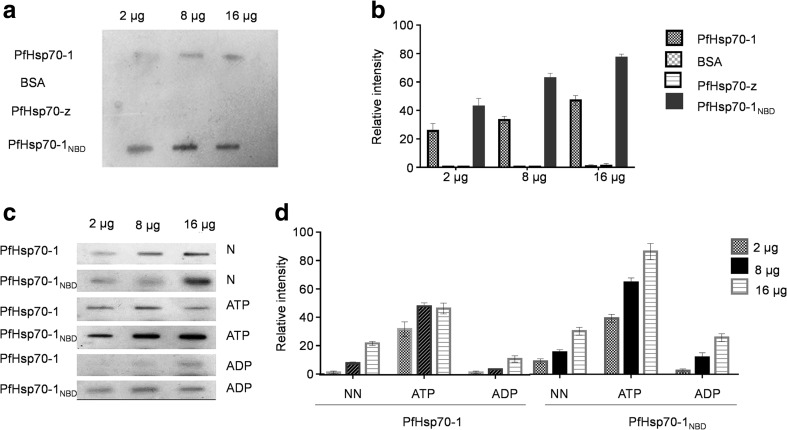
Nucleotide-dependent interaction of PfHsp70-z and PfHsp70-1. Slot blot analysis (**a**) and accompanying densitometric analysis (**b**) confirming the specificity of anti-PfHsp70-1 antibodies; slot blot analysis (**c**) and accompanying densitometric analysis (**d**) demonstrating the interaction of PfHsp70-z with PfHsp70-1/PfHsp70-1_NBD._ The assay was conducted in the absence of nucleotide (*NN*) or presence of 5 μM ATP/ADP, respectively. The reported data was obtained from at least three independent assays conducted using different protein batches. *Error bars* are indicated. Dose-dependent interaction between PfHsp70-z and PfHsp70-1 was ascertained by densitometric analysis using ANOVA (*p* < 0.001)

To further validate the association of PfHsp70-z and PfHsp70-1, we conducted a slot blot assay using the recombinant forms of the respective proteins. Prey protein (PfHsp70-z) was immobilized onto the blot, and the bait protein (PfHsp70-1/PfHsp70-1_NBD_) and control protein (BSA) were passed over the immobilized PfHsp70-z protein. This was followed by conducting immunoblotting using anti-PfHsp70-1 antibodies (Shonhai et al. 2008). The association of the two proteins was further assessed in the presence of ATP/ADP. We previously validated that anti-PfHsp70-1 antibodies do not cross-react with PfHsp70-z (Zininga et al. 2015a). To further validate the specificity of the anti-PfHsp70-1 antibodies, we immobilized full length PfHsp70-1 and PfHsp70-1_NBD_ at various concentrations onto the nitrocellulose membrane along with PfHsp70-z and BSA as control. The subsequently conducted immunoblot analysis confirmed that the antibodies were specific for PfHsp70-1 and PfHsp70-1_NBD_ (Fig. 8a). Densitometric analysis of the blot suggested that anti-PfHsp70-1 antibodies possess higher affinity for the truncated form of the protein (PfHsp70_NBD_) than full length PfHsp70 (Fig. 8b). Furthermore, in the absence of nucleotide and in the presence of ADP/ATP, PfHsp70-z interacts with both full length PfHsp70-1 and its NBD subdomain in a dose-dependent fashion (Fig. 8c, d). As previously observed for the immunoaffinity and coaffinity chromatography assays, ATP appeared to enhance the association of the two proteins compared to ADP or absence of nucleotide. These findings suggest that the PfHsp70-1_NBD_ is the primary site through which PfHsp70-z interact with PfHsp70-1. Furthermore, since this interaction occurs in the presence of ATP, it is unlikely that the association is based on chaperone-substrate interaction since PfHsp70-1 releases its substrates in the presence of ATP (Shonhai et al. 2008). In addition, the observed interaction between the NBD subdomain of PfHsp70-1 and PfHsp70-z further suggests that the association is not based on chaperone (PfHsp70-1)-substrate (PfHsp70-z)-based interaction. It is possible that PfHsp70-z may have acted as a chaperone binding mis-folded PfHsp70-1. However, we rule out this possibility since PfHsp70-1 is known to be stable and functional even at high temperatures (Shonhai et al. 2008). It is therefore unlikely that a significant amount of this protein would have been misfolded. In addition, we purified the protein, maintaining it in native form throughout the purification process. Furthermore, prior to conducting the heat-induced aggregation suppression assays, we subjected both proteins to heat stress at 48 °C and established that none of them aggregated under these conditions (data not shown). Taking all these aspects into consideration, we rule out the possibility that PfHsp70-z may have bound to PfHsp70-1 in a chaperone/substrate association. Therefore, our findings suggest that PfHsp70-z interacts with PfHsp70-1, possibly by establishing ionic contacts with specific residues located in the NBD of the latter.

**Fig. 9:**
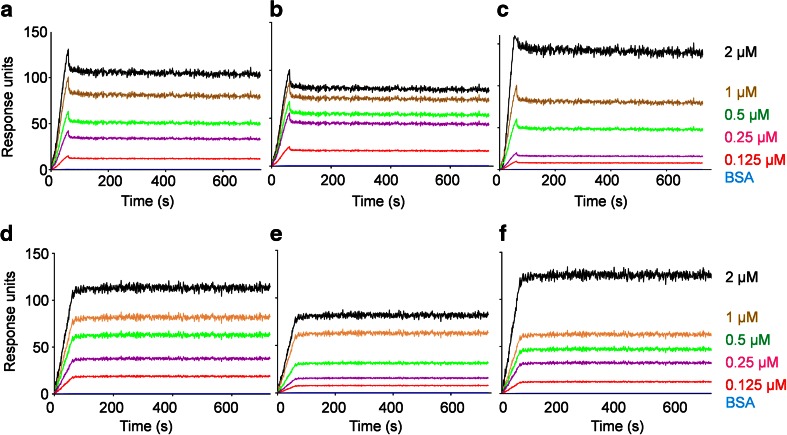
PfHsp70-z directly interacts with PfHsp70-1/PfHsp70-1_NBD_. SPR sensorgrams for the interaction of PfHsp70-z with PfHsp70-1/PfHsp70-1_NBD_. The interaction between PfHsp70-1 and PfHsp70-z (**a**); the experiment was repeated in the presence of 5 mM ADP (**b**); and 5 mM ATP (**c**). The interaction of PfHsp70-z and PfHsp70-1_NBD_ (**d**); the experiment was repeated in the presence of 5 mM ADP (**e**); and 5 mM ATP (**f**). The association exhibits concentration dependence with respect to levels of analyte protein used. The data also shows nucleotide-dependent association. The detailed binding kinetics generated are summarized in Table 2

We also investigated the interaction kinetics of the two proteins using SPR based on a simple Langmuir fit model (Fig. 9). We investigated the interaction of PfHsp70-z with either full length PfHsp70-1 (Fig. 9a–c) or its NBD subdomain (Fig. 9d–f). The array system allowed for analysis of all the proteins, alternating as either ligands or analytes. Overall, the SPR kinetic data further confirmed that the two proteins interact in the absence and presence of either ATP or ADP (Table 2). The presence of ATP enhanced the affinity of PfHsp70-z and PfHsp70-1 (full length or ATPase domain) compared to the affinity observed in the presence of ADP or absence of nucleotide. This is consistent with the previous observations we made based on immunoaffinity (Fig. 7) and slot blot (Fig. 8)-based assays.

**Table 2:** Binding kinetics of the interaction between PfHsp70-z and PfHsp70-1

Ligand, analyte	Nucleotide	*k*_a_ (Ms^−1^)	*k*_d_ (s^−1^)	*K*_D_ (M)	*χ* ^2^
PfHsp70-1, PfHsp70-z	ATP	9.97 (±0.8) e+02	2.42 (±0.1) e-05	2.41 (±0.2) e-08*	1.86
	ADP	2.98 (±0.2) e+02	3.61 (±0.3) e-05	1.21 (±0.1) e-05	2.12
	–	4.95 (±0.1) e-01	2.96 (±0.2) e-05	5.98 (±0.5) e-05	3.32
PfHsp 70-z, PfHsp70-1_NBD_	ATP	4.46 (±1.1) e+03	9.43 (±0.6) e-06	2.12 (±0.2) e-09*	2.65
	ADP	2.63 (±1.3) e+03	2.60 (±0.5) e-04	9.86 (±0.9) e-08	6.52
	–	2.39 (±0.6) e+00	3.76 (±0.2) e-05	1.57 (±0.1) e-08	2.44

The interaction kinetics represented by the association rate constant (*k*_a_), dissociation rate constants (*k*_d_), and equilibrium constant (*K*_D_) were determined by SPR analysis alternating the status of PfHsp70-z and PfHsp70-1/PfHsp70-1_NBD_ as ligand and analyte, respectively. The ligand was the respective immobilized protein on the GLC chip surface, and the analyte was the respective protein injected at a flow rate of 50 μl/min. Data were analyzed by using readings obtained for the running buffer (in the presence of nucleotides but in the absence of protein analyte) as baseline (Fig. 9). Data are represented as mean plus/minus standard deviation. Chi-square (*χ* ^2^) values represent the Langmuir curve fitting residuals. The statistical analysis was conducted using one way ANOVA

*p < 0.005, statistically significant differences in affinities noted under the variable experimental conditions

## Discussion

PfHsp70-z is an essential molecule which localizes to the parasite cytosol and is thought to inhibit aggregation of malarial asparagine repeat-rich proteins (Muralidharan et al. 2012). This protein is thus thought to mask the effects of stress that the parasite encounters in the host. In addition, we previously demonstrated that PfHsp70-z is an ATPase and that is it a heat-stable molecule (Zininga et al. 2015a). Our current findings represent the first evidence showing that PfHsp70-z possesses independent chaperone function. Furthermore, we established that PfHsp70-z directly interacts with PfHsp70-1 in a nucleotide-dependent fashion. In addition, we observed that this interaction occurs primarily through the N-terminal ATPase domain of PfHsp70-1. We speculate that PfHsp70-z may associate with PfHsp70-1 in order to facilitate the latter’s nucleotide exchange function. It has been suggested that PfHsp70-z and PfHsp70-1 are both expressed at the clinical phase of malaria, and their expression pattern is reported to mirror malaria pathology (Pallavi et al. 2010). We think that the two proteins work in tandem to facilitate protein quality control in the parasite.

PfHsp70-z is predicted to be a NEF of PfHsp70-1 (Shonhai et al. 2007). In order to carry out this function, PfHsp70-z must be able to bind nucleotides. We therefore enquired if the conformation of PfHsp70-z would be modulated by nucleotides. We employed partial proteolysis (Liberek et al. 1991) to elucidate the interaction of PfHsp70-z with nucleotides. Since PfHsp70-z possesses two tryptophan residues, we further conducted tryptophan fluorescence-based analysis to validate our findings. We observed that PfHsp70-z released fragments exhibiting similar size profiles when it was digested in the absence of nucleotide or in the presence of ADP. This suggests that nucleotide-free and its ADP-bound forms assume conformations that are nearly identical. This was corroborated by the fluorescence-based analysis (Fig. 5a). This is in agreement with a previous study which suggested that nucleotide-free Hsp110 assumes a similar conformation to its ADP-bound state (Raviol et al. 2006). On the other hand, in the presence of ATP, PfHsp70-z appears to assume a unique conformation based on both limited proteolytic and tryptophan fluorescence analyses. The ATP-bound form of PfHsp70-z was most resistant to proteolysis compared to nucleotide-free/ADP-bound forms of the protein. This suggests that PfHsp70-z is protected from trypsin cleavage by conformational changes induced by ATP. This is in agreement with previous study which reported that human and yeast Hsp110 both assumed a compact conformation upon binding ATP (Raviol et al. 2006).

Hsp110 proteins exhibit unique linker segments that connect the N-terminal NBD to the C-terminal SBD (Polier et al. 2008). It has been suggested that the linker of Hsp110 is fairly dynamic, allowing the protein to exhibit some degree of allosteric function (Liu and Hendrickson 2007). Based on sequence alignment, plasmodial Hsp110 proteins exhibit a linker segment that is distinct from that of canonical Hsp70s. Furthermore, the linker segment of PfHsp70-z is divergent from that of human and yeast Hsp110s. Since the linker region is required for Hsp70 allosteric function (Vogel et al. 2005), we surmise that the linker of plasmodial Hsp110 is unique and may thus confer specific functional features to these proteins. Indeed, it has been proposed that Hsp110 from yeast may possess unique structural features compared to the human homologue (Raviol et al. 2006). It is thus plausible that PfHsp70-z may possess functional features that make it unique from its human counterpart. The unique structure-function features of plasmodial heat shock proteins have facilitated their selective targeting as potential drug targets (Shonhai 2010; Cockburn et al. 2011). Hsp70s exhibit less conserved substrate binding domains. Because PfHsp70-z possess a longer and less conserved substrate binding domain, it presents a more promising option for selective targeting compared to canonical Hsp70s such as PfHsp70-1 whose substrate binding domain is shorter.

Based on nucleotide binding assays, we could not ascertain whether ATP binding by PfHsp70-z resulted in global structural conformation or whether the conformational changes were restricted to the NBD only. However, since PfHsp70-z was able to suppress heat-induced aggregation of protein in the absence and presence of nucleotides, we think that the protein may possess limited allosteric function. On the other hand, the holdase chaperone function of PfHsp70-1 is sensitive to ATP binding (Shonhai et al. 2008; Fig. 6). In light of this, we surmise that PfHsp70-z possesses a more rigid linker segment than that of PfHsp70-1. It is plausible that a rigid linker may confer PfHsp70-z with better substrate holding function than that of PfHsp70-1. Indeed, our current findings suggest that PfHsp70-z was marginally better at suppressing heat-induced aggregation of protein in vitro (Fig. 6). It has been estimated that nearly one quarter of *P. falciparum* proteome are thought to possess asparagine repeat-rich regions which are prone to aggregation under heat stress (Singh et al. 2004). In addition, malaria parasites survive under hostile conditions in the host which are characterized by cyclic fever phases. It is therefore conceivable that the parasites require a robust molecular chaperone system to deal with the protein quality demands. Our findings suggest that PfHsp70-z plays an important role in the survival of malaria parasites through its function as a holdase chaperone which maintains proteostasis. This is in agreement with a previous study which proposed that PHsp70-z suppresses aggregation of plasmodial asparagine-rich proteins (Muralidharan et al. 2012). PfHsp70-1 also exhibits protein aggregation suppressive functions (Shonhai et al. 2005, 2008, this study). We therefore think that in this regard, PfHsp70-1 supports PfHsp70-z in suppressing protein misfolding and aggregation in *P. falciparum*. However, since PfHsp70-1 is sensitive to ATP binding and possesses a functional linker segment, it is likely to serve both as a holdase and refoldase chaperone. On the other hand, PfHsp70-z whose linker segment is divergent from that of canonical Hsp70s may not facilitate allosteric communication between the ATPase domain and the substrate binding domain. This restriction may limit its direct function as a chaperone to protein aggregation suppression.

Data obtained from the tryptophan fluorescence assay showed that the conformation of PfHsp70-z was responsive to the presence of ATP (Fig. 5a). However, nucleotides (ATP/ADP) did not influence the chaperone function of PfHsp70-z with respect to suppression of protein aggregation. Furthermore, our findings suggest that ATP promotes the interaction of PfHsp70-z with PfHsp70-1. Since only ATP, and not ADP, regulates the conformation of PfHsp70-z, it is likely that the influence of ATP on the association of PfHsp70-z with PfHsp70-1 could be due to the combined effect of the nucleotide on the conformations of both proteins. Thus, ATP possibly forces the two proteins to individually assume conformations that favor their association. It is also possible that the effect of ATP on the conformation of PfHsp70-z is restricted to localized perturbation of the nucleotide binding domain. This would allow the protein bind substrate equally effectively in the presence of ATP as in the absence of nucleotide or presence of ADP.

We further established that PfHsp70-1_NBD_ constitutes the minimum subdomain required for interaction with PfHsp70-z. It is thought that the ATP-bound form of Hsp110 is capable of directly binding the NBD of canonical Hsp70 to facilitate ADP release from the latter (Polier et al. 2008). We therefore propose that one of the key functions of PfHsp70-z is to serve as NEF of PfHsp70-1. Inhibition of PfHsp70-1 is associated with parasite death (Chiang et al. 2009), and it has been proposed that this protein could constitute an antimalarial drug target (Shonhai 2010; Cockburn et al. 2011, 2014; Zininga and Shonhai 2014). According to genomic data, *P. falciparum* does not possess a cytosol-localized GroP-like gene E (GrpE) homologue, neither has a Bag-1 or HspBP1 homologue been identified as a potential NEF of PfHsp70-1. Therefore, PfHsp70-z is likely to be the sole NEF of PfHsp70-1.

Altogether, our findings suggest that apart from its role as a molecular chaperone, PfHsp70-z may participate in a broad spectrum of functions, which could include facilitating the nucleotide exchange function of PfHsp70-1. We think that PfHsp70-z and PfHsp70-1 work in tandem to facilitate protein quality control in the parasite. While PfHsp70-1 may exhibit functional versatility through facilitating both protein folding and preventing protein aggregation, we think that PfHsp70-z is more robust at suppressing protein aggregation. The fact that the expression of both of these proteins is reported to mirror the progression of clinical malaria (Pallavi et al. 2010) suggests that their partnership is important for the development of malaria parasites.
